# Design and characterization of quad port dual band MIMO antenna for 5G V2X connectivity

**DOI:** 10.1038/s41598-026-44515-3

**Published:** 2026-03-18

**Authors:** Sriram Arumugam, Sangeetha Manoharan, Mohamed Abraar Abbas, I. M. Suraaj, Sachin Kumar, Tanweer Ali

**Affiliations:** 1 Department of Electronics and Communication Engineering, SRM Institute of Science and Technology, Kattankulathur, 603203 India; 2 Department of Electronics and Communication Engineering, Galgotias College of Engineering and Technology, Greater Noida, 201310 India; 3 Manipal Institute of Technology, Manipal Academy of Higher Education, Manipal, India

**Keywords:** Engineering, Physics

## Abstract

This paper proposes a quad-port dual-band multiple-input-multiple-output (MIMO) antenna that operates at the n78 band (3.5 GHz) and the dedicated short-range communications band (5.9 GHz), addressing the requirements of emerging 5G and vehicle-to-everything (V2X) communication systems. The presented MIMO antenna employs multi-stub radiating structures with a defected ground configuration using planar, non-resonant geometries without vias and multilayer stacking to achieve compact footprint of 0.95*λ*_*g*_ × 0.95*λ*_*g*_, improved impedance matching and enhanced bandwidth without integrating substrate integrated waveguide cavities, complementary split ring resonators and neutralization line techniques. The fabricated antenna prototype offers measured impedance bandwidths of 680 MHz and 670 MHz at both operating bands, with mutual coupling less than −20 dB. The antenna has a gain of 3.6 dBi at 3.5 GHz and 4.7 dBi at 5.9 GHz, while maintaining an efficiency of more than 83% and an omnidirectional radiation pattern suitable for vehicular integration. MIMO diversity analysis shows an envelope correlation coefficient < 0.03, diversity gain ≈ 9.99 dB, total active reflection coefficient < -10 dB, and channel capacity loss ≈ 0.1 bps/Hz, indicating excellent diversity and isolation performance. Furthermore, the antenna performance is validated on the issue of housing effect and the vehicle installation environment, whereby roof top integration increases the directivity while maintaining the omnidirectional coverage, demonstrating that it is suitable for reliable, high data rate 5G-enabled V2X communication.

## Introduction

The continuous evolution of wireless communication technologies propelled by 5G innovations and rising demand for vehicle-to-everything (V2X) connectivity, brings the development of advanced antenna designs to provide link reliability, low latency and high-speed communication^1^. The frequency band of 3.5 GHz (3.4–3.6 GHz, n78 band) is dedicated for broad coverage and rise of intelligent transportation systems (ITS) operating at 5.9 GHz (5.850–5.925 GHz), are evolving traction due to their compatibility with 5G infrastructure^2^. Moreover, the advancement of multiple-input-multiple-output (MIMO) improved the data carrying capacity through spatial multiplexing without compromising bandwidth requirement^3^. Many antenna types have been proposed for V2X applications over the years, such as shark fin antennas, which combine multiple independent antennas operating at different frequencies on the roof of the car^4–7^. But these antennas fail to meet essential performance criteria, including adequate isolation and optimal radiation pattern characteristics. The spiral antennas proposed in^5,6^ for V2X communication do not provide adequate link reliability in the vehicular environment. In^7^, the authors proposed a wheel-shaped fractal vehicular antenna, which does not support the n78 band for 5G communication.

Several studies have focused on MIMO antenna design by combining several antenna radiators into a single module suitable for integration in vehicular communication^8^, with the goal of overcoming key challenges such as compactness, isolation and maintaining stable radiation patterns to improve link reliability in the most robust environments. In^9^, the authors proposed a 4-port diversity antenna that works in the n78 and n96 bands, with multiple strips as radiator and defected ground structure (DGS). One of the most important parameters to consider in MIMO antenna design is isolation between adjacent antenna elements. To improve the isolation, several techniques have been used, including the neutralization techniques^10,11^ and the complementary split ring resonator (CSRR)^12^. In^13^, the authors used coplanar waveguide (CPW) feed configuration to design MIMO antenna with improved isolation between the antenna elements, whereas^14^ proposes a meandered line fed CPW antenna to cover the Wi-Fi/Wi-MAX/5G bands. A metamaterial-based CPW antenna proposed in^15^ can operate at 5.2 GHz, 5.8 GHz and 5.5 GHz frequencies. In^16,17^, the authors presented MIMO antennas with element organized orthogonally to provide better isolation for dedicated short-range communications (DSRC) applications. In^2^, the authors designed a quad-port MIMO antenna for 5G and V2X applications using a rectangular patch with metamaterial loading, achieving maximum gains of 2.9 dBi and 3.7 dBi, respectively.

An antenna with multiple microstrip line radiating elements proposed in^18^ resonates at the n77, n78 and n96 bands, with a marginal gain of 0.576 dBi in the n96 band. In^19^, an eight-element array antenna measuring 136 mm × 68 mm is used to cover the LTE, n77, n78 and n79 bands. The antenna described in^20^ operates in the 3.3–5.95 GHz range and uses an 8-element MIMO configuration with parasitic strip lines and a DGS to achieve mutual coupling below −15 dB. In contrast, the antenna design described in^21^ accommodates both the n78 and DSRC frequency bands. In^22^, a cavity-backed quad-port antenna using the substrate integrated waveguide (SIW) technique is introduced to work at the n78 band with 1.39 dBi gain. The quad-port MIMO antenna designed in^23^ uses three distinct bands of 2.9 GHz, 5.0 GHz and 5.9 GHz, with two substrates embedded on each other via photolithography, making the antenna setup complex. Quad-port MIMO antennas in^24,25^ covers the DSRC band for V2X communication and uses DGS etching to suppress surface current. In^26,27^, the authors proposed inverted L-shaped radiators and a perturbed hexagonal patch for quad-port MIMO antennas that can operate in the n78 and DSRC bands for 5G V2X communication. In^28^, a quad-port MIMO antenna with frequency selective surface (FSS) structure for 5G wireless communication is reported, with a substrate thickness of 12.5 mm and an antenna gain of 6.7 dBi. In^29^, inkjet-printed silver onto 3D-printed polypropylene is used to design a dual-band slotted patch antenna for wearable devices. A high-gain ultra-wideband antenna with a slotted partial ground plane has been reported in^30^, where the gain of the antenna is enhanced from 4 dBi to 8.3 dBi using FSS as a reflector. While the above-mentioned antenna designs improve isolation significantly, the use of stubs, SIW and CSRR-loaded elements tends to increase structural complexity and overall antenna size.

This paper presents a dual-band quad-port diversity antenna with DGS. Initially, a single element dual-band antenna is designed to resonate at the n78 and DSRC bands, and to improve diversity performance in harsh environments, the single element antenna is transformed into a quad element configuration arranged horizontally and vertically. The elements are positioned orthogonally without any decoupling structure, resulting in improved channel capacity and link dependability suitable for 5G-enabled vehicular applications. Further, the proposed antenna is investigated on the roof top of a vehicle (3D CAD model) in the free space environment to analysis its far-field property.

The main features of the proposed quad-port dual-band MIMO antenna are as follows:

- The antenna supports dual-band frequency ranges of 3.5 GHz (n78 band) and 5.9 GHz (DSRC band), with measured impedance bandwidths of 680 MHz in n78 band and 670 MHz in DSRC band. This allows for 5G enabled automotive communication with minimal radiating elements.
- Several reported MIMO antenna designs employ complex isolation enhancement techniques such as SIW cavities^20^, CSRR^2^, DGS^23^ and neutralization lines^9,24^. In contrast, the proposed antenna achieves strong isolation (> 20 dB) and very low envelope correlation coefficient (ECC) (< 0.03) without any additional decoupling structures, relying solely on the orthogonal configuration of radiating elements. This significantly reduces electromagnetic and fabrication costs when compared to complex decoupling-based MIMO structures while maintaining excellent diversity performance in complex multipath environments with polarization diversity.
- The proposed quad-port MIMO antenna achieves a channel capacity loss (CCL) of ≈ 0.1 bps/Hz, total active reflection coefficient (TARC) < −10 dB, and a diversity gain (DG) of ≈ 9.99 dB, even without a decoupling structure, making it a suitable candidate for vehicular environments.
- The analysis of the antenna’s rooftop installation on a vehicle demonstrates reliable far-field characteristics.

The paper is structured as follows: The section “Design and analysis of dual-band antenna” describes the design of a single element dual-band antenna based on multiple stub integration and explains the operation of each structure. Section “Quad-port dual-band MIMO antenna design” describes the design of a quad-port diversity antenna and shows various experimental analyses on diversity characteristics with an antenna prototype, while section “Investigation of the proposed dual-band antenna on vehicle body” investigates the far-field analysis. Lastly, the key findings are summarized in the section “Conclusion”.

## Design and analysis of dual-band antenna

### Antenna element

**Fig. 1:**
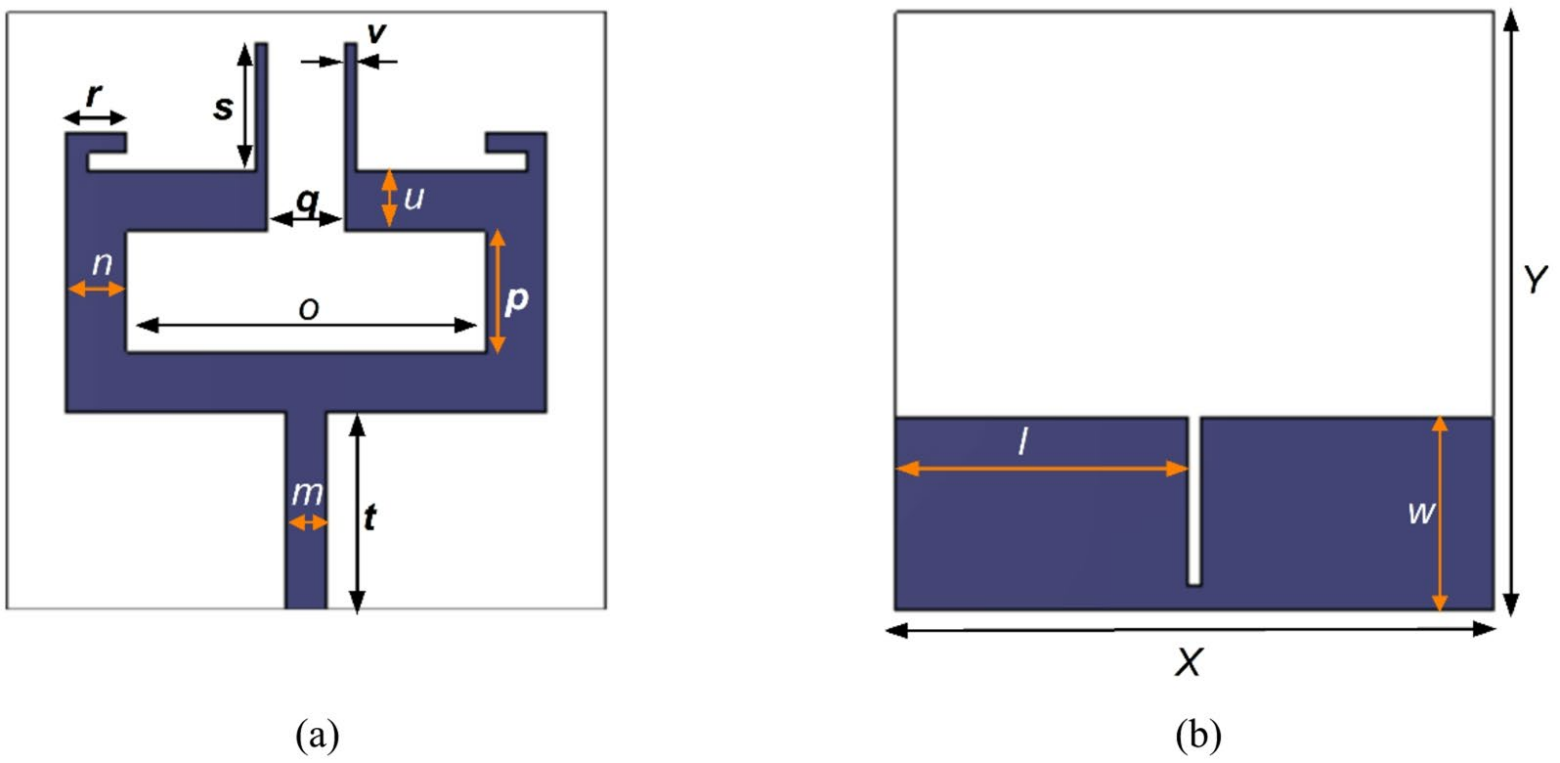
Dual-band antenna with structural dimensions: **a** Front view, **b** Back view.

Figure 1 depicts the layout of the proposed dual-band antenna element. The antenna is designed on the Rogers substrate (RO3003) with dielectric constant of 3, a thickness of 0.13 mm and a loss tangent of 0.001. The top layer features a multi-stub radiating structure whereas the bottom layer integrates a defected ground configuration to enhance performance. A 50 Ω microstrip feed line is used to excite the antenna. The dimensions of the designed multiband antenna are determined using Eqs. (1) and (2), where *c* denotes the speed of light.

1 $${f}_{3.5GHz}=\frac{0.21*c}{\left(t+\left(\frac{p}{2}\right)+o+u\right)*\sqrt{\frac{{\epsilon}_{r}+1}{2}}}$$

2 $${f}_{5.9GHz}=\frac{0.11*c}{\left(t+\left(\frac{p}{2}\right)+o+u+s\right)*\sqrt{\frac{{\epsilon}_{r}+1}{2}}}$$

**Fig. 2:**
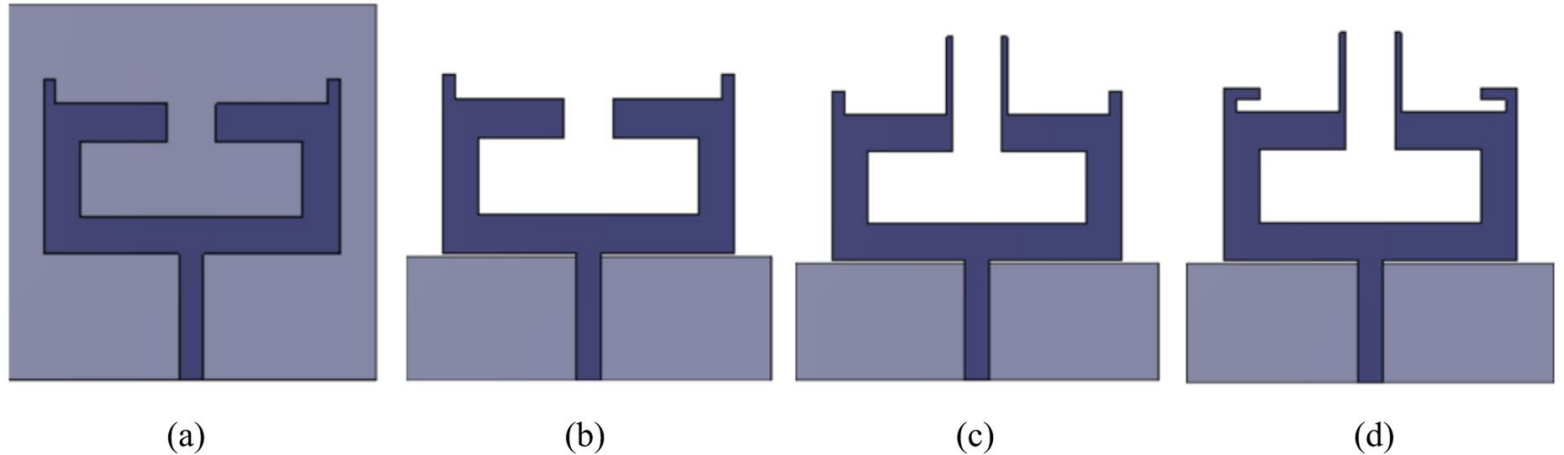
Development of dual-band antenna: **a** Stage 1, **b** Stage 2, **c** Stage 3, **d** Stage 4.

**Fig. 3:**
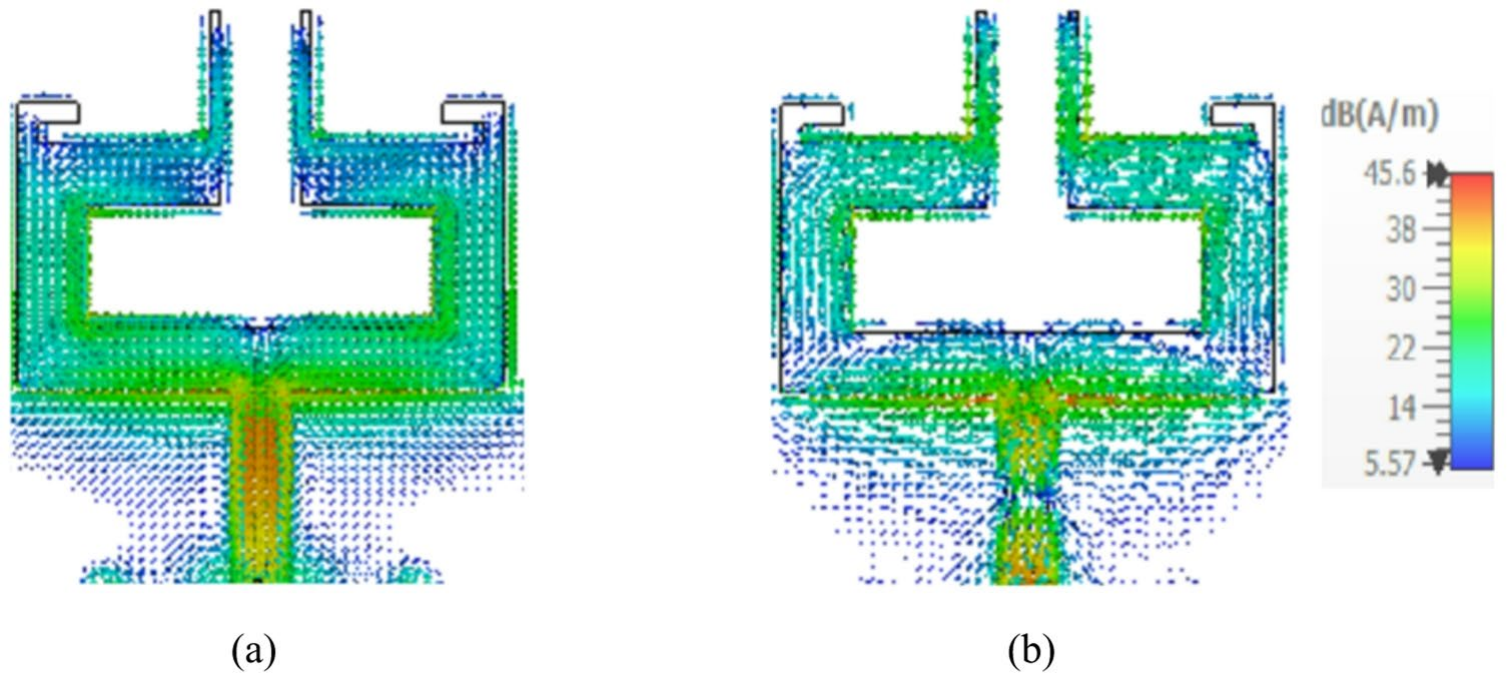
Field distribution of fundamental dual-band antenna: **a** 3.5 GHz, **b** 5.9 GHz.

**Fig. 4:**
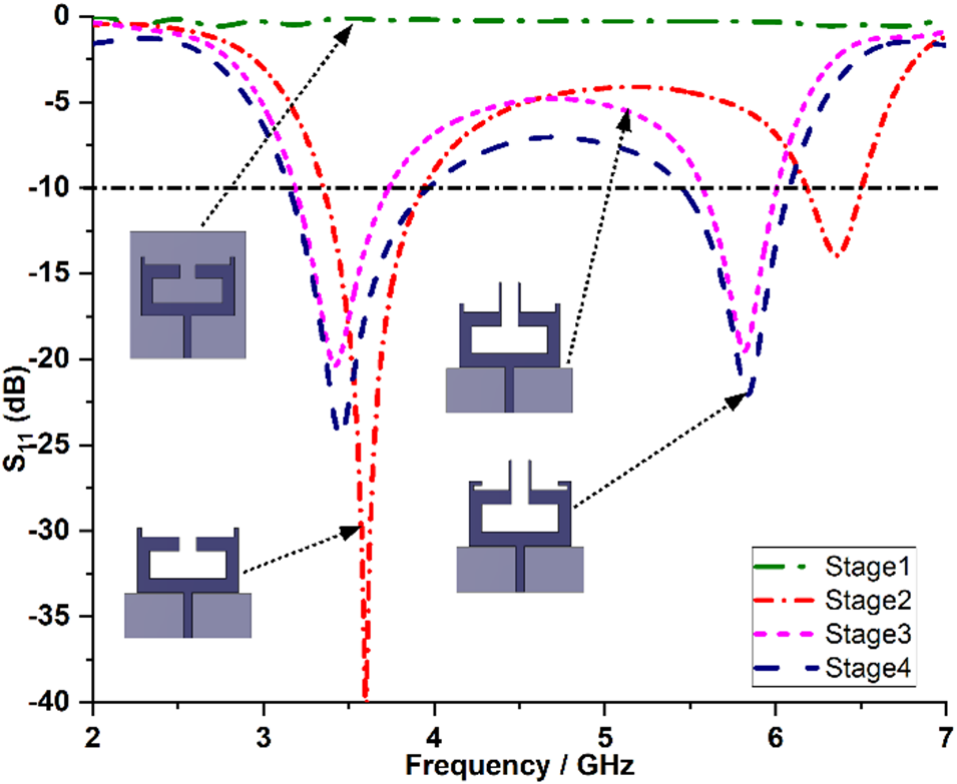
Reflection coefficients of design stages of the proposed dual-band antenna.

The design dimensions of the antenna are: *m* = 2 mm, *n* = 3 mm, *o* = 18 mm, *p* = 6 mm, *q* = 4 mm, *r* = 3 mm, *s* = 6.5 mm, *t* = 10 mm, *u* = 3 mm, *v* = 0.5 mm, *w* = 9.75 mm, *l* = 14.7 mm, *X* = 30 mm and *Y* = 30 mm. Figures 2 and 3 illustrate the sequential design evolution and field distribution of the proposed antenna element, respectively. Initially, the antenna is configured in a compact rectangular configuration as shown in Stage 1 (Fig. 2(a)) incorporating a complete ground plane. However, this structure demonstrates limited impedance bandwidth with S_11_ values remaining above −10 dB as indicated in Stage 1 of Fig. 4. To improve impedance characteristics, the continuous ground plane is modified into a defected ground, as presented in Stage 2 (Fig. 2(b)). This modification facilitates dual resonances at 3.35 to 3.9 GHz and 6.18 to 6.5 GHz offering bandwidths of 550 MHz and 320 MHz, respectively, as depicted in Stage 2 of Fig. 4. Also, a strong field intensity is observed along the outer edges of the rectangular stub (on both left and right sides) at 3.5 GHz as depicted in Fig. 3(a).

Furthermore, in order to achieve resonance at the second harmonic frequency of 5.9 GHz, a vertical stub radiator is incorporated at the centre of the top layer in Stage 3 (Fig. 2(c)), which exhibits a strong field concentration along the longer edges of the vertical stub radiator at 5.9 GHz, as illustrated in Fig. 3(b). This addition provides inductive loading, improving the Q-factor as expressed in Eq. (3) and enhance the overall impedance bandwidth. Consequently, the antenna achieves an additional resonance within the 5.57 to 6.0 GHz range, offering a bandwidth of 630 MHz, as shown in Stage 3 of Fig. 4.

3 $$Q=\frac{2fL}{R}=\frac{{X}_{L}}{R}$$

To achieve optimal impedance matching, a small horizontal stub is added on both the left and right sides of the antenna in Stage 3, resulting in the modified configuration shown in Stage 4 (Fig. 2(d)). This adjustment enables frequency coverage from 3.15 to 3.9 GHz and 5.4 to 6.1 GHz, corresponding to bandwidths of 750 MHz and 700 MHz, respectively, as illustrated in Stage 4 of Fig. 4.

### Parametric analysis of the single element dual-band antenna

**Fig. 5:**
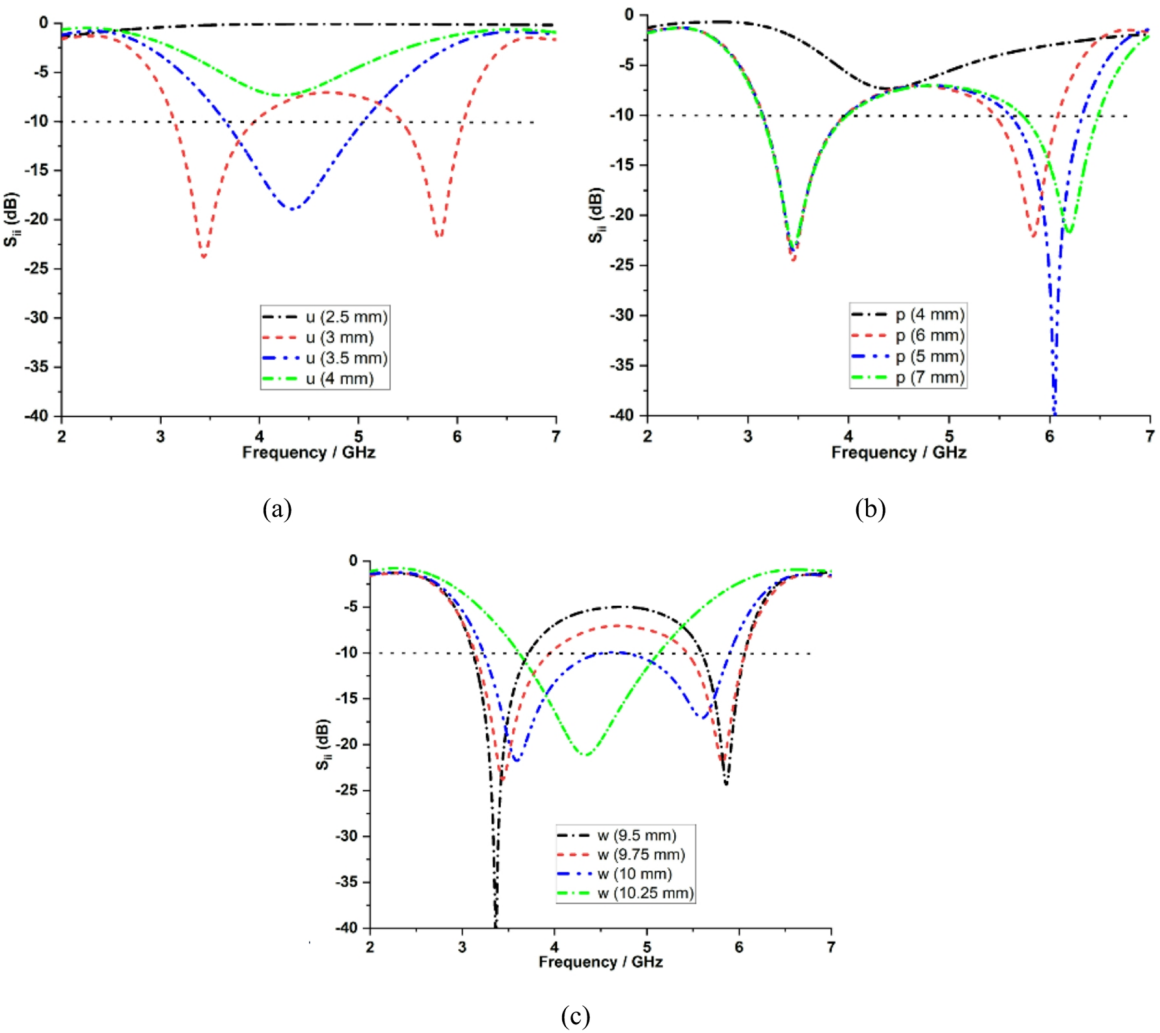
Variation in the: **a** upper horizontal strip line ($u$), **b** vertical strip line ($p$), **c** ground plane length ($w$)..

A parametric analysis of the key design parameters has been included to illustrate their impact on the impedance characteristics of the proposed dual-band antenna. The analysis investigates the effects of the upper horizontal strip line ($u$), vertical strip line ($p$) and ground plane length ($w$). Figure 5(a) depicts the variation in the upper horizontal strip length (*u*). The value of *u* primarily affects the lower resonant band at 3.5 GHz by acting on the path of the predominant mode current. When *u* is increased, the effective electrical length increases, and the resonance shifts above −10 dB, resulting in poor impedance matching. The value of *u* is set to 3 mm, which resonates at 3.5 GHz and provides an acceptable impedance matching.

Figure 5(b) shows the variation of the vertical strip line (*p*), which primarily shifts the upper resonant band at 5.9 GHz by altering the shorter current path of the higher-order mode. A good impedance is achieved by setting the *p* value to 6 mm at 5.9 GHz. Figure 5(c) depicts the influence of the ground plane length (*w*) on both operating bands. The optimal value of *w* = 9.75 mm is used to achieve stable dual-band operation with improved impedance matching.

**Fig. 6:**
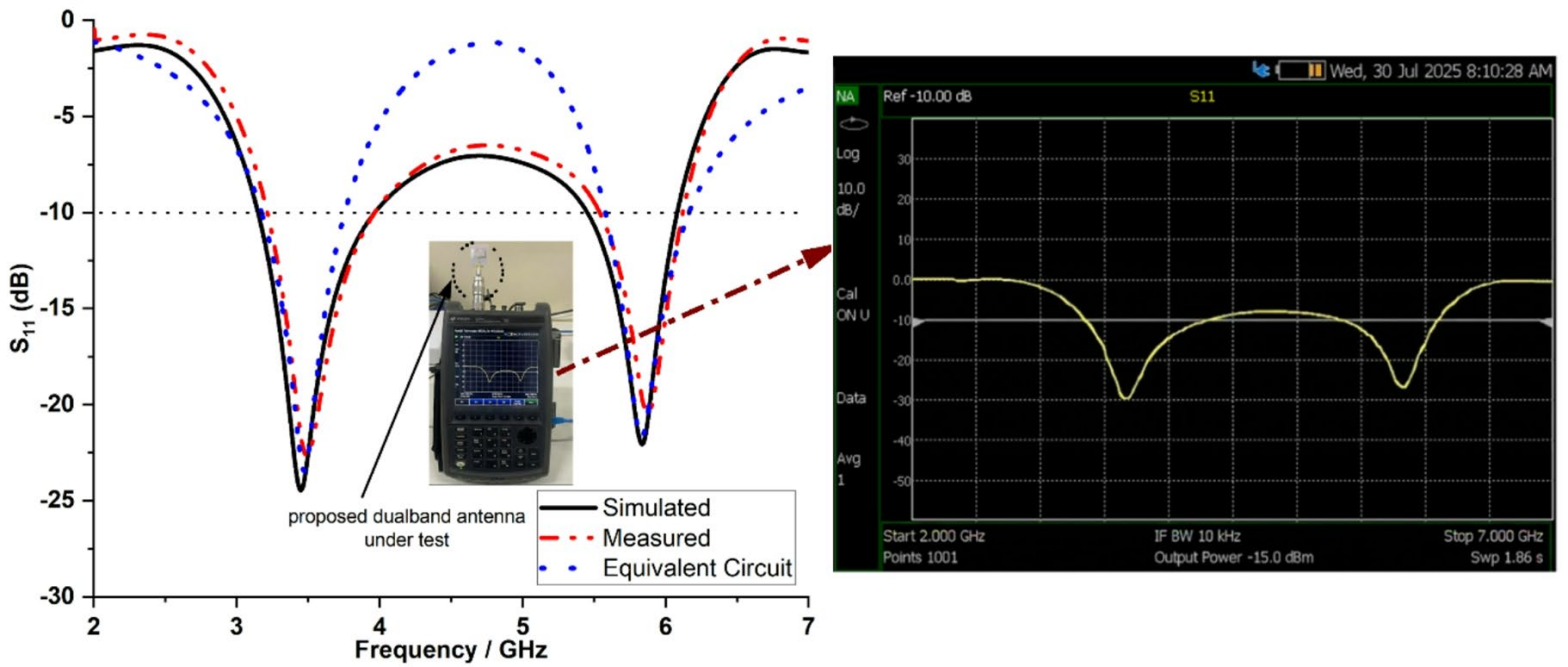
Simulated and measured reflection coefficients of the proposed dual-band antenna.

Figure 6 shows the measured reflection coefficients taken with the Anritsu MS2037C vector network analyser to validate the proposed antenna prototype. The antenna operates in two bands: 3.5 GHz and 5.9 GHz, covering frequencies from 3.2 to 3.88 GHz and 5.5 to 6.17 GHz, respectively with a measured impedance bandwidth of 680 MHz and 670 MHz.

**Fig. 7:**
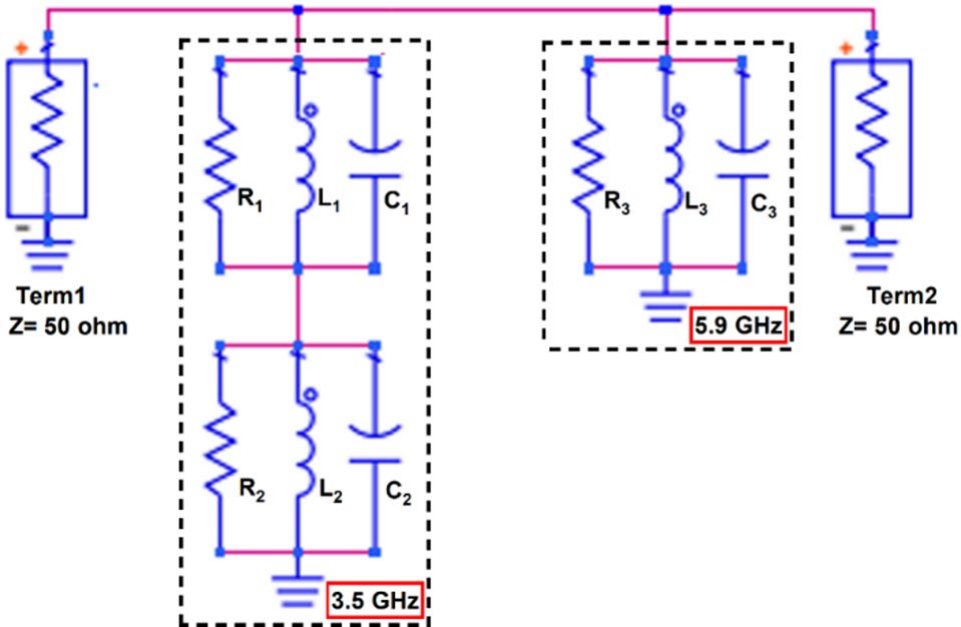
Equivalent circuit of the proposed dual-band antenna.

Figure 7 depicts the equivalent circuit of the proposed dual-band single element antenna resonating at 3.5 GHz and 5.9 GHz, with the values listed in Table 1. The circuit representation explains the antenna geometry, the physical origin of the observed resonances and validates antenna behavior through its correspondence with the simulated results. The proposed antenna is terminated with 50 Ω matched elements that resonate at different frequencies, indicating a different mode of operation. The lumped elements *R*_2_, *L*_2_ and *C*_2_ represent the fundamental mode of an electrical long current path along the main radiator and the horizontal rectangular stub at 3.5 GHz. At the fundamental resonance frequency, the components *L*_2_ and *C*_2_ provide excellent impedance matching. The elements *R*_3_, *L*_3_ and *C*_3_ correspond to the higher order mode of an electrical short current path along the vertical stub radiator that operates at 5.9 GHz. Meanwhile, the components *R*_2_ and *R*_3_ represent conductor (dielectric) and radiation losses. The resonating elements *R*_1_, *L*_1_ and *C*_1_ are added by the small horizontal radiating stubs on top, increases the parasitic coupling effect and bandwidth. The capacitors *C*_2_ and *C*_3_ model the fringing and radiator ground field coupling. The equivalent circuit for the proposed antenna is consistent with its dual-band impedance matching capability. Figure 6 shows the reflection coefficient characteristics of the proposed equivalent circuit at 3.5 GHz and 5.9 GHz. The slight discrepancies among the simulated, equivalent circuit and measured results can be attributed to approximations inherent in the lumped element modelling, manufacturing tolerances and the effects of connector.

**Table 1:** RLC values of the equivalent circuit.

Frequency (GHz)	Resister (Ω)		Inductor (nH)		Capacitor (pF)	
3.5	*R*_1_	5000	*L*_1_	1.65	*C*_1_	0.08
	*R*_2_	1060	*L*_2_	7.9	*C*_2_	0.07
5.9	*R*_3_	2900	*L*_3_	0.08	*C*_3_	1.85

**Fig. 8:**
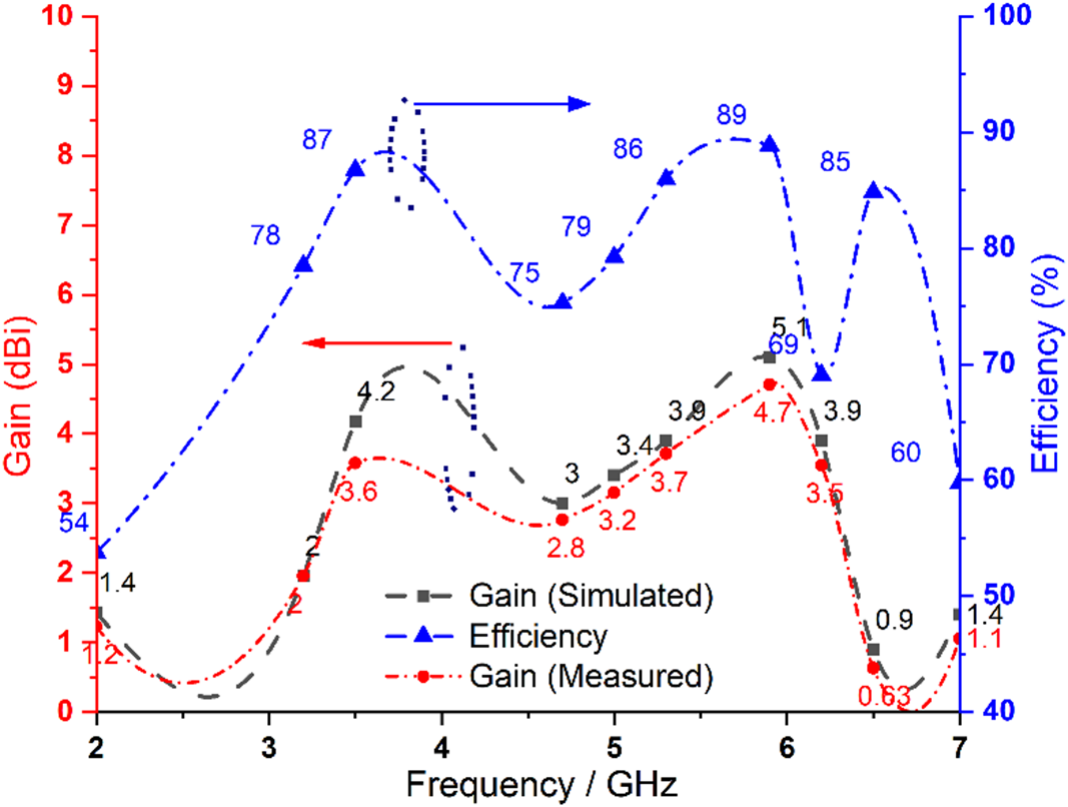
Gain and radiation efficiency of the proposed dual-band antenna.

**Fig. 9:**
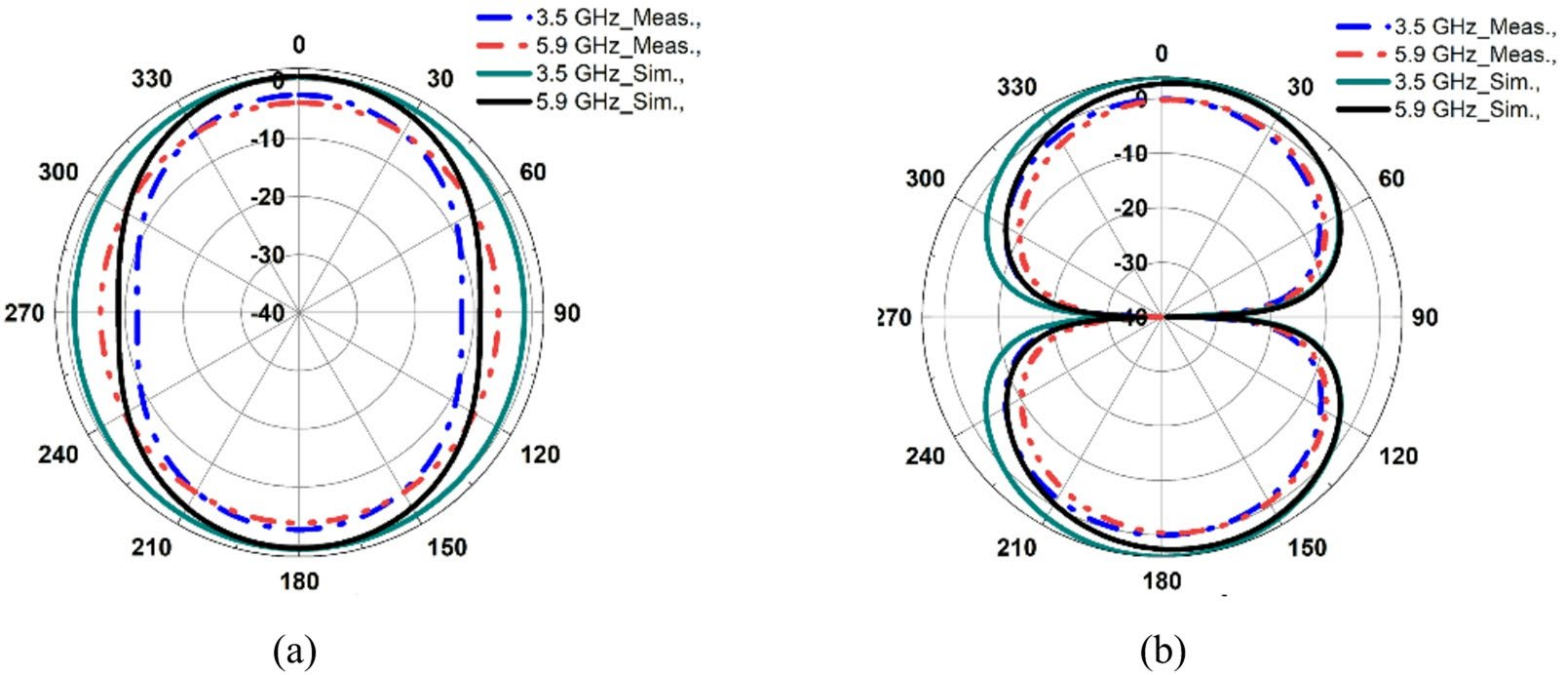
Far-field characteristics of the proposed dual-band antenna: **a** Phi = 0˚, **b** Phi = 90˚.

Figure 8 illustrates the variation of gain and efficiency of the proposed dual-band antenna element. The antenna achieves an efficiency exceeding 83% in both operating bands of 3.5 GHz and 5.9 GHz. The maximum simulated gain values are 4.2 dBi at 3.5 GHz and 5.1 dBi at 5.9 GHz while the corresponding measured gain values are 3.6 dBi and 4.7 dBi, respectively. The proposed dual-band antenna exhibits an omnidirectional radiation pattern at both operating frequencies ensuring uniform coverage, as depicted in Fig. 9.

### Quad-port dual-band MIMO antenna design

**Fig. 10:**
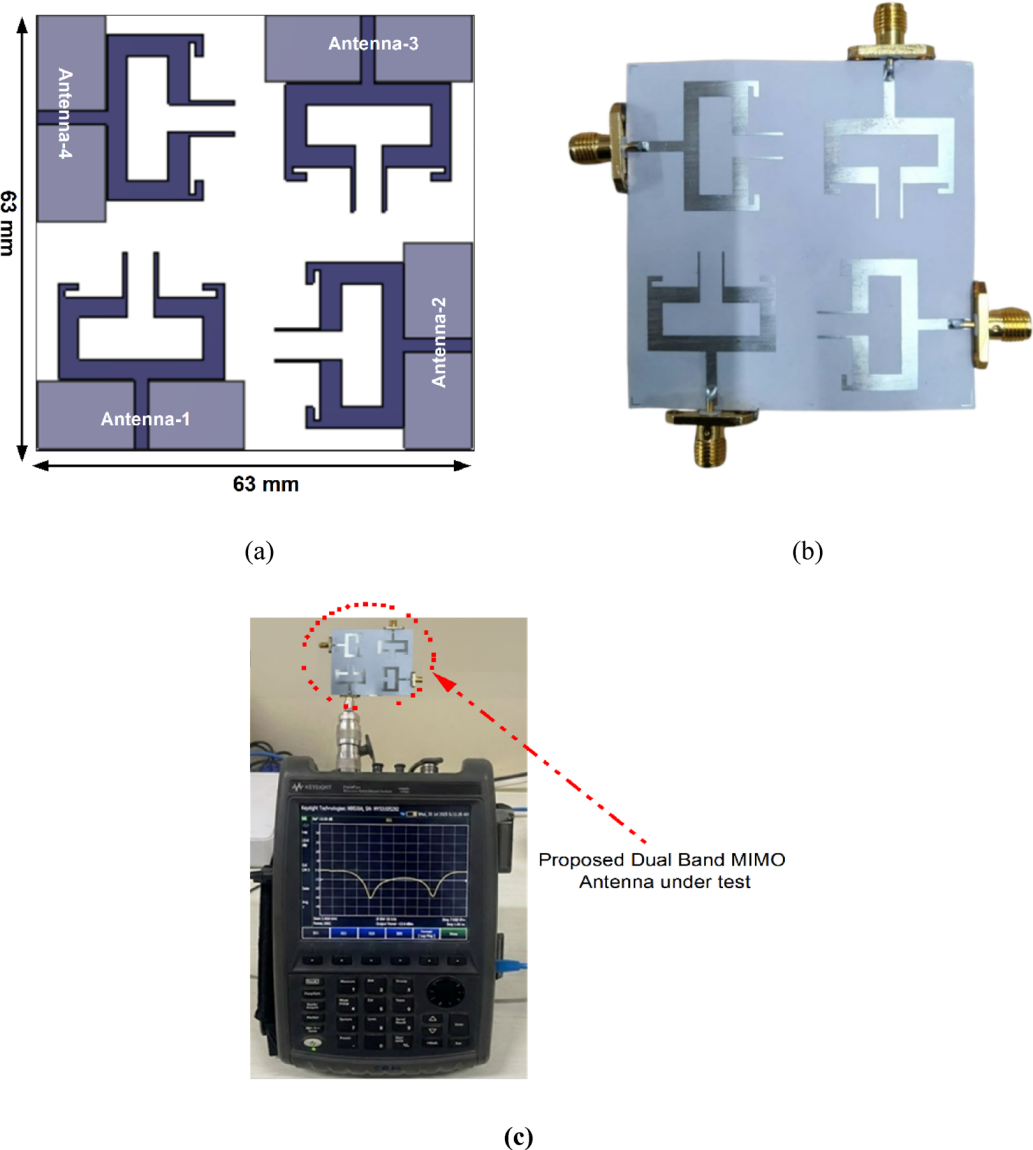
Configuration of the proposed MIMO antenna, **b** Fabricated MIMO antenna prototype. **c** MIMO antenna measurement setup on VNA.

The quad-port MIMO/diversity antenna is designed to achieve higher data rates, improved link reliability and reduced fading effects in multipath environments, thereby improving overall diversity performance. Figure 10 shows that four identical dual-band antenna elements are arranged orthogonally to ensure effective signal reception under diversity conditions. The elements are separated by 7 mm, resulting in a total antenna dimension of 63 mm × 63 mm. The configured and fabricated prototype of the proposed quad-port dual band MIMO antenna is shown in Fig. 10.

**Fig. 11:**
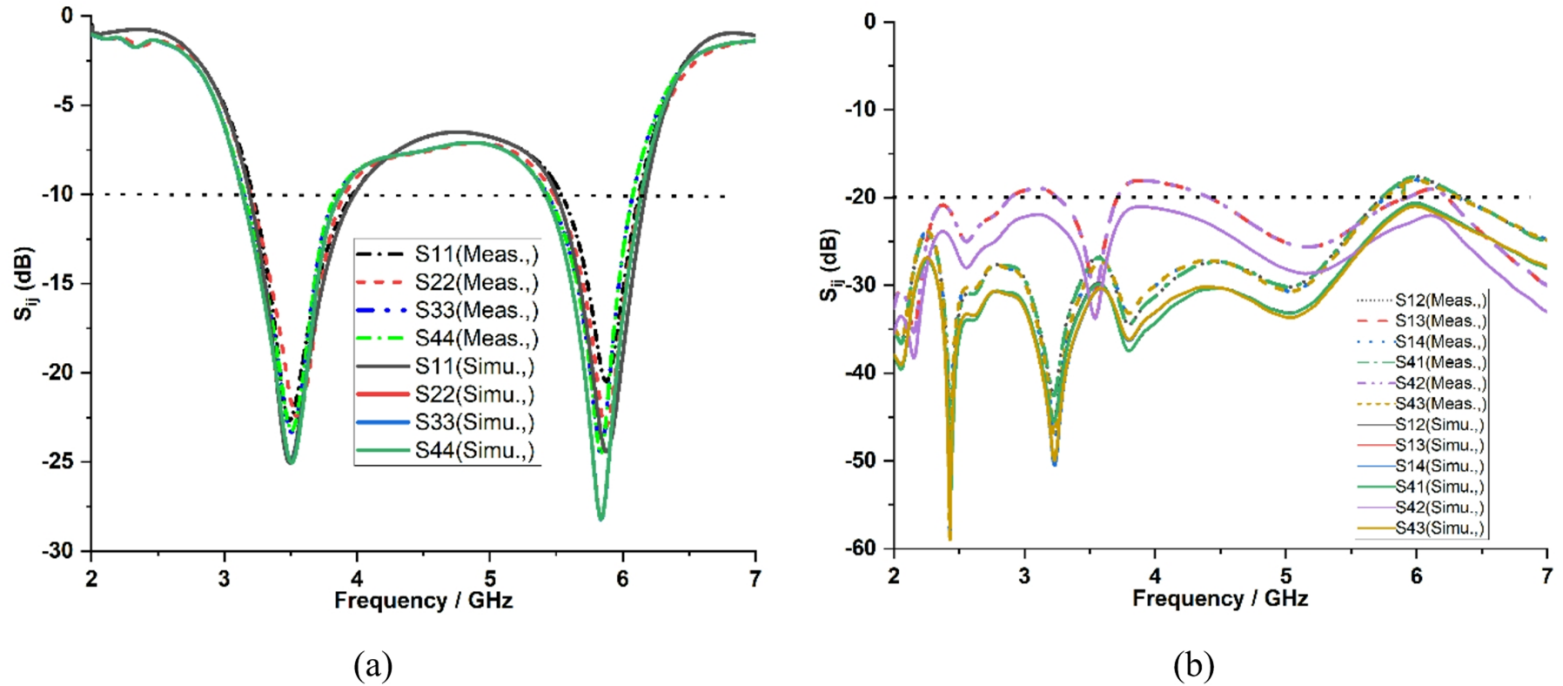
**a** Simulated and measured reflection coefficients (S₁₁, S₂₂, S₃₃, S₄₄) of the antenna, **b** Mutual coupling between antenna ports (S₁₂, S₁₃, S₁₄, S₄₁, S₄₂, S₄₃).

The simulated and measured S-parameter results of the proposed design are presented in Fig. 11. The proposed MIMO antenna shows measured impedance bandwidth of 680 MHz and 670 MHz in both operating bands from 3.2 to 3.88 GHz and 5.5 to 6.17 GHz corresponding to the center frequencies of 3.5 GHz and 5.9 GHz as shown in Fig. 11(a). Furthermore, the measured mutual coupling between all antenna ports remains below −20 dB in both frequency bands with the smallest interelement spacing, confirming good isolation as shown in Fig. 11(b). This is achieved by without integrating any neutralization lines^10^, CSRR-based loading^12^, parasitic loading or decoupling elements^20,21^, SIW cavities^22^, multilayer stacking^23^ and band-stop structures, which are normally employed to enhance isolation but create more complex to design and more sensitive to fabrication. The symmetrical design of the structure provides identical performance characteristics for port 1 and port 4.

**Fig. 12:**
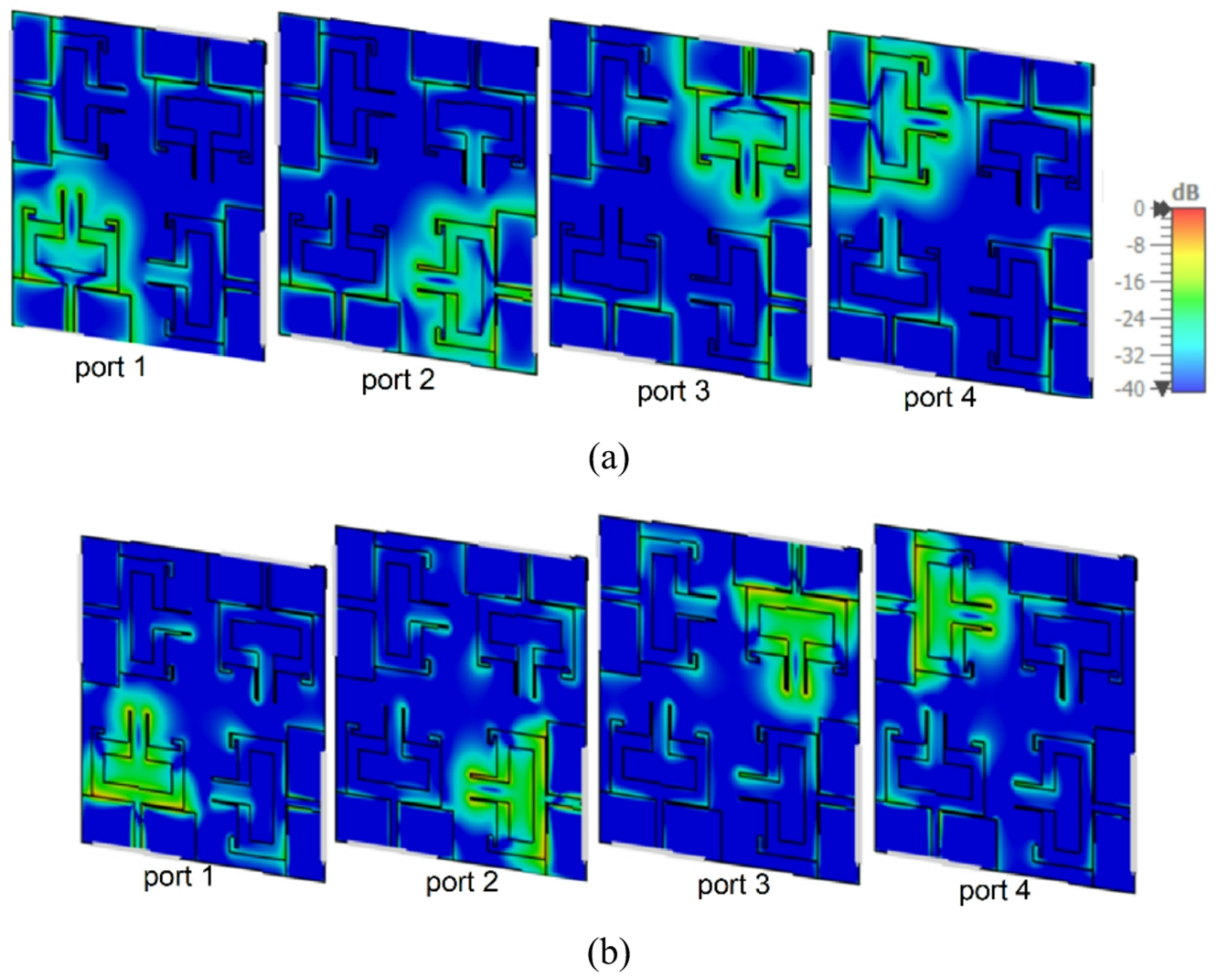
Surface current distributions of the proposed MIMO antenna: **a** 3.5 GHz, **b** 5.9 GHz.

Figure 12(a) and (b) depict the simulated surface current distributions of the proposed quad-port MIMO antenna at 3.5 GHz and 5.9 GHz when each antenna is activated independently while other antennas are connected with matched loads. When port 1 of the MIMO antenna is excited at 3.5 GHz and 5.9 GHz, more current distribution remains on the rectangular radiating element along the meandered line and slot loaded radiators of the patch connected to port 1. This confirms that the current peaks are more on resonant paths, and the antenna resonates in its fundamental resonant mode and higher order resonant mode at these frequencies. Meanwhile, the nearby antennas have weak coupling currents (port 2 to port 4) with good electromagnetic isolation. The same reaction occurs when ports 2, 3 and 4 are excited one at a time, as shown in Fig. 12(a) and (b).

**Fig. 13:**
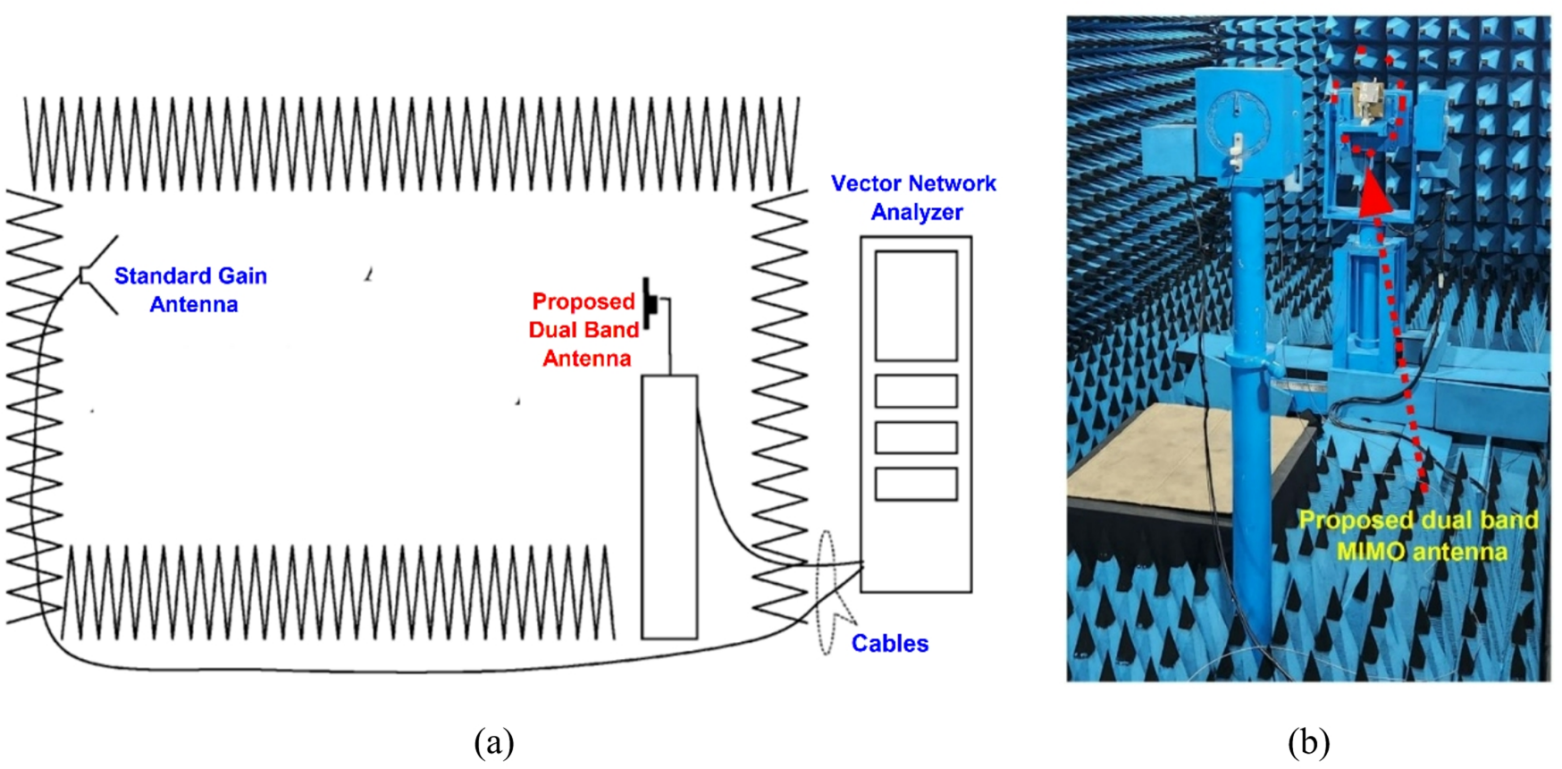
**a** Antenna measurement setup, **b** MIMO antenna measurement setup in the anechoic chamber.

The proposed dual-band antenna is characterized under far-field conditions by keeping a distance of 3 m between the standard antenna and the proposed antenna at 3.5 GHz and 5.9 GHz, respectively, to meet the far-field requirements as shown in Fig. 13(a).

**Fig. 14:**
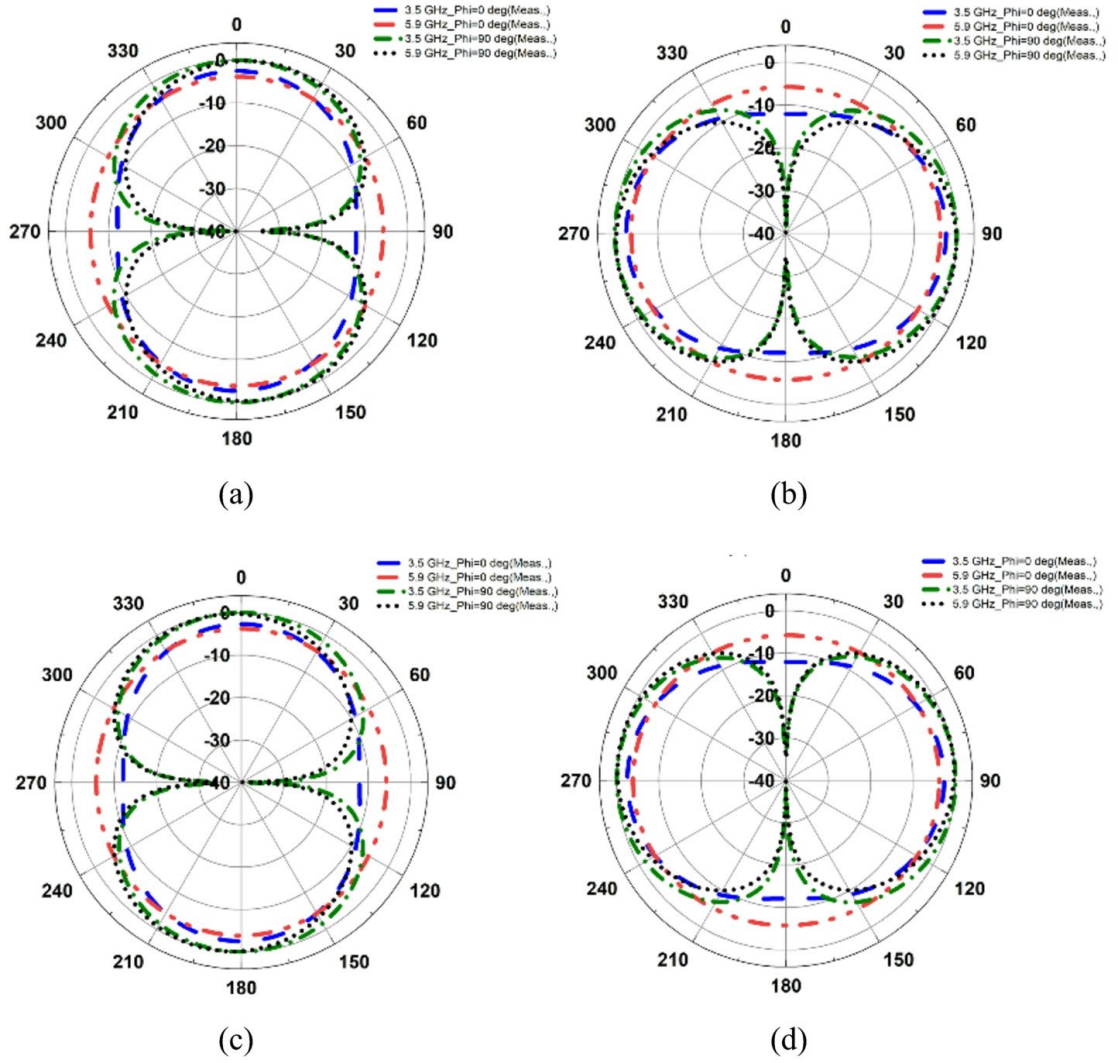
Measured far-field characteristics at n78 and DSRC bands: **a** Antenna 1, **b** Antenna 2, **c** Antenna 3, **d** Antenna 4.

**Fig. 15:**
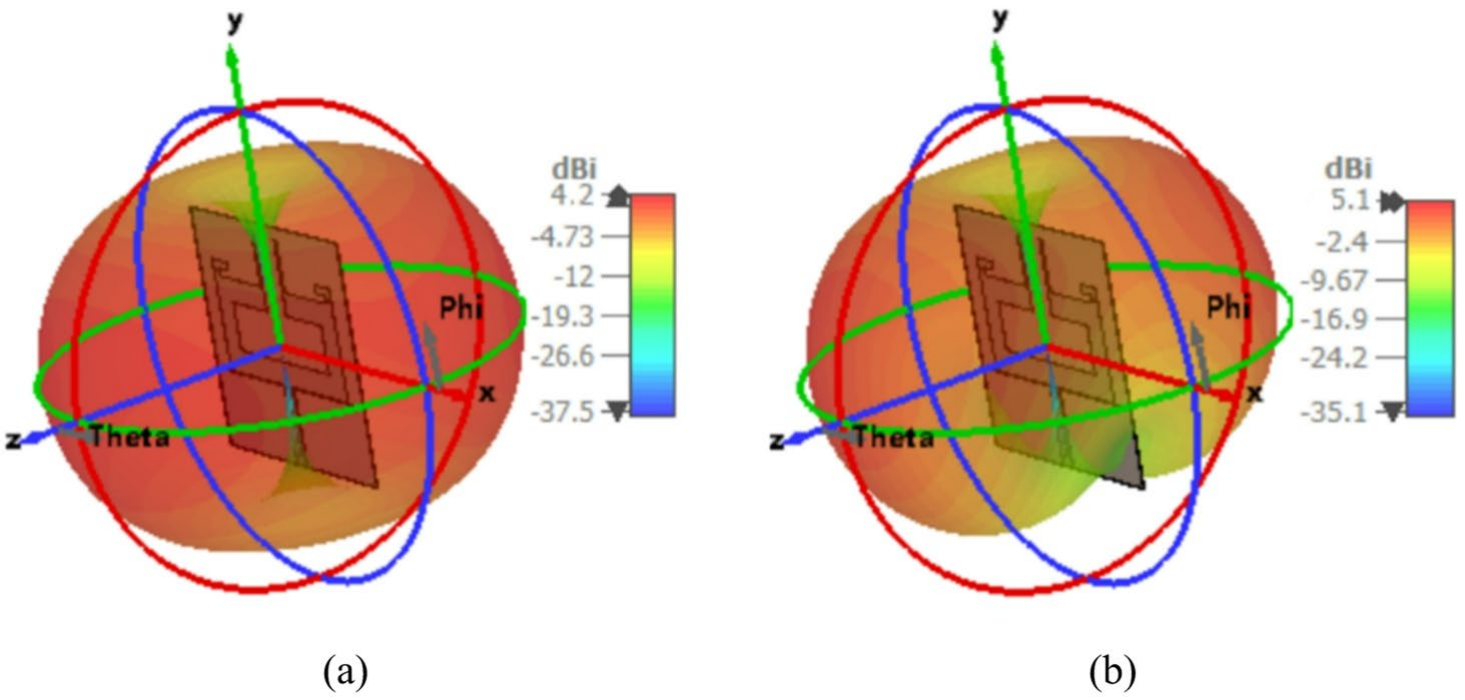
3D patterns of the proposed dual-band antenna: **a** 3.5 GHz, **b** 5.9 GHz.

The fabricated MIMO antenna is evaluated for *φ* = 90° (E-plane) and *φ* = 0° (H-plane) by rotating the proposed dual-band antenna, as illustrated in Fig. 13(b). The results show that the proposed antenna has an omnidirectional radiation pattern at both frequencies, as shown in Fig. 14, resulting in improved signal coverage and reliable operation in diversity communication environments. Figure 15 shows the corresponding 3D radiation patterns of the proposed dual-band antenna at 3.5 and 5.9 GHz (antenna 1 is shown because the remaining antennas are symmetrical).

**Fig. 16:**
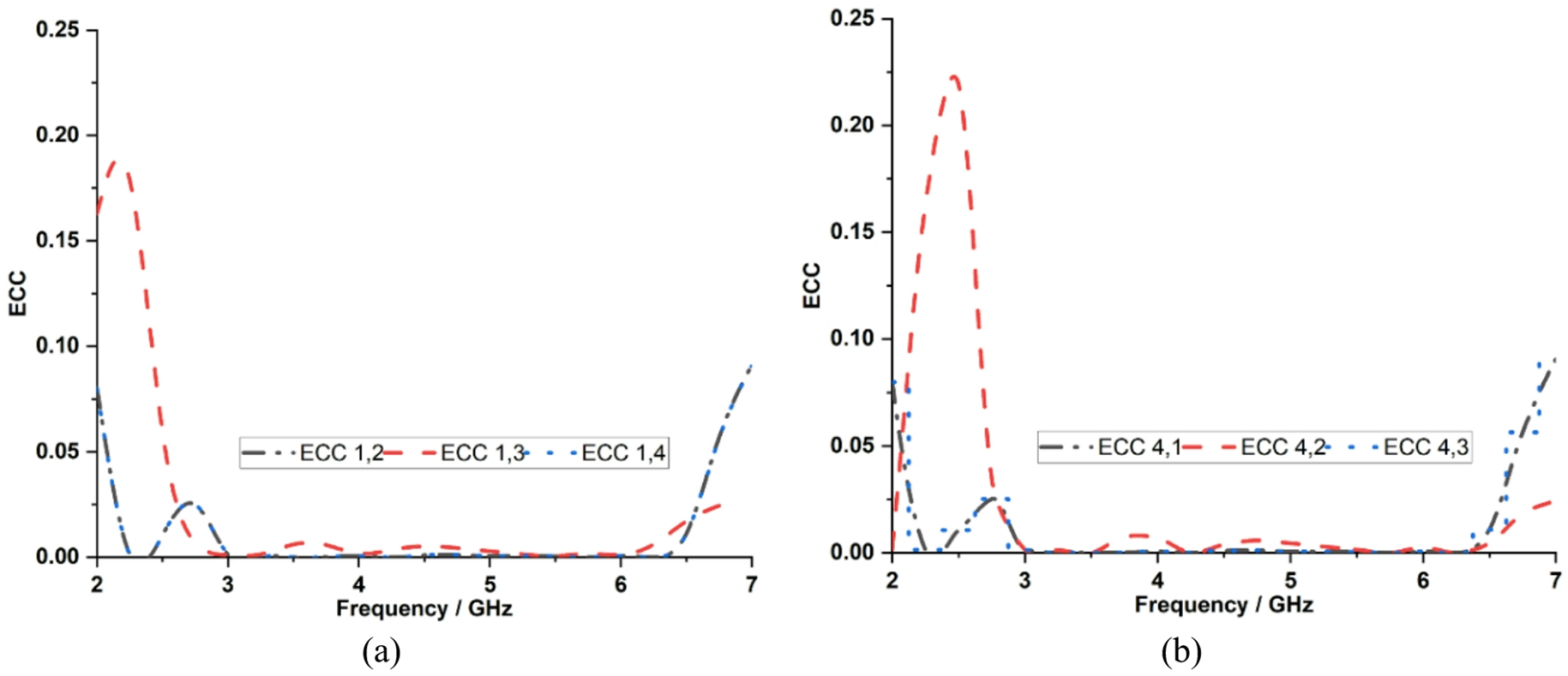
Measured ECC characteristics using the S-parameter with respect to **a** Port 1, **b** Port 4.

**Fig. 17:**
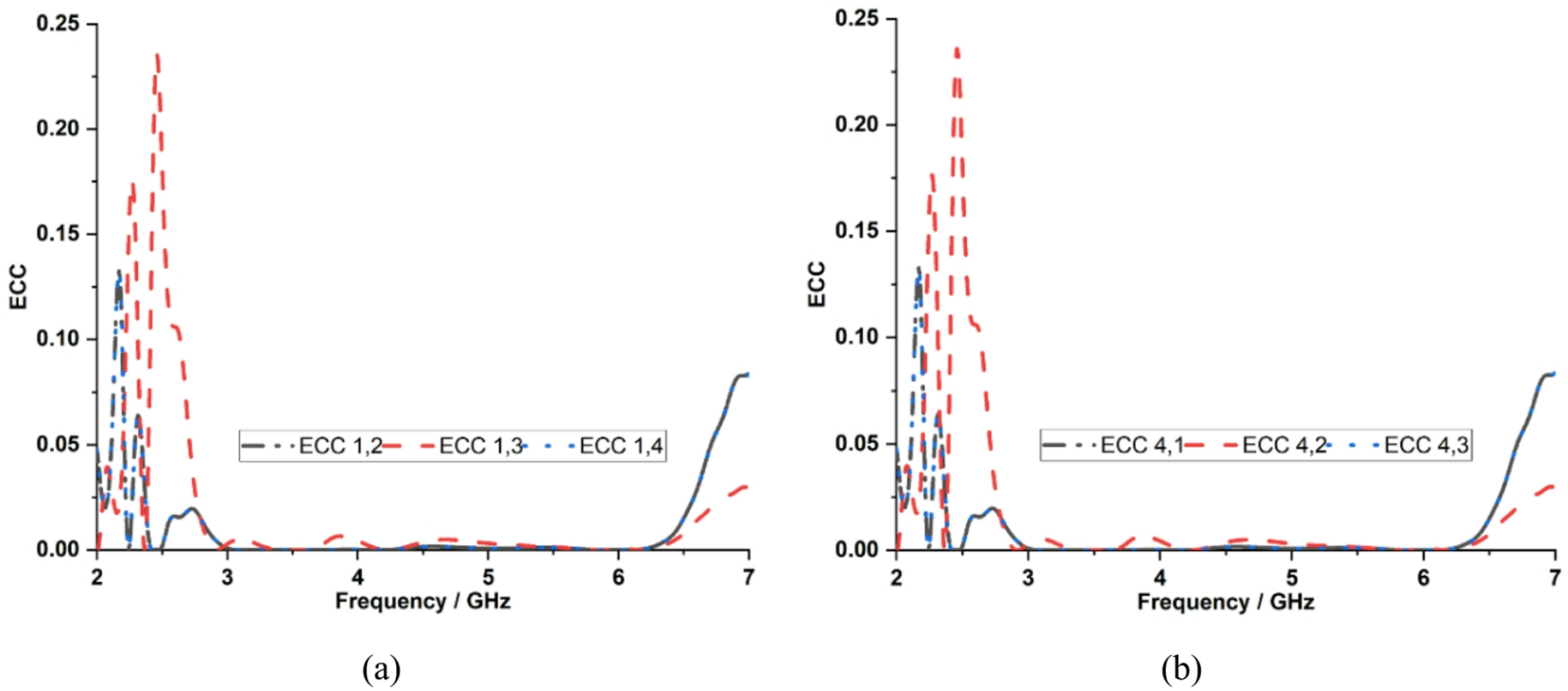
Measured ECC characteristics using far-field with respect to **a** Port 1, **b** Port 4.

ECC is an important parameter in evaluating diversity performance because it quantifies the level of correlation between radiation characteristics or mutual coupling between MIMO antenna elements. ECC can be calculated using the S-parameter and the far-field as shown in Eqs. (4) and (5). Figure 16 shows that using the S-parameter, the measured ECC values for the proposed dual-band MIMO antenna are 0.007 and 0.002 at 3.5 and 5.9 GHz, respectively. Figure 17 shows ECC less than 0.03 at 3.5 GHz and 5.9 GHz using far-field, indicating excellent diversity performance due to effective surface current confinement on the excited elements, as well as reduced electromagnetic interaction with an optimal interelement spacing of 7 mm. However, the ECC calculation based on S-parameter is not concerned with antenna losses and is limited by its accuracy, which is less than 50% of the radiation efficiency^31^.

4 $${\rho}_{e}=\frac{{\left|{S}_{ii}^{*}{S}_{ij}+{S}_{ji}^{*}{S}_{jj}\right|}^{2}}{(1-{\left|{S}_{ii}\right|}^{2}-{\left|{S}_{ji}\right|}^{2})(1-{\left|{S}_{jj}\right|}^{2}-{\left|{S}_{ij}\right|}^{2})}$$

5 $${\rho}_{e}=\frac{\left|\iint{E}_{1}\left(\theta,\phi\right).{E}_{2}^{*}(\theta,\phi\right)sin\theta d\theta d\varnothing{|}^{2}}{\left(\iint{\left|{E}_{1}(\theta,\phi)\right|}^{2}sin\theta d\theta d\varnothing\right)\left(\iint{\left|{E}_{2}(\theta,\phi)\right|}^{2}sin\theta d\theta d\varnothing\right)}$$

where ${E}_{1}\left(\theta,\phi\right)and{E}_{2}\left(\theta,\phi\right)$ are the complex 3D radiation patterns of two antenna elements.

**Fig. 18:**
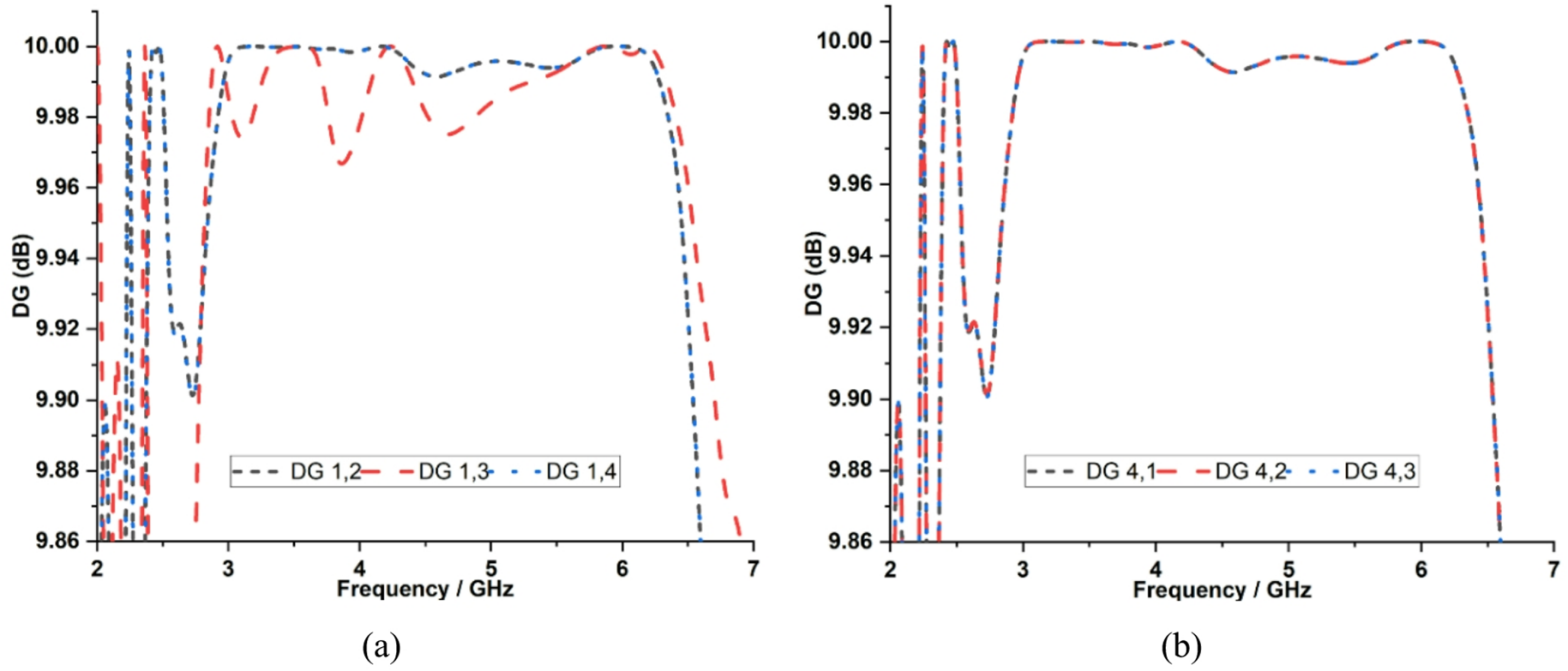
Measured DG characteristics with respect to **a** Port 1, **b** Port 4.

The DG of the proposed quad-port MIMO antenna is calculated using Eq. (6), which provides a value of 9.99 dB at both 3.5 GHz and 5.9 GHz, as shown in Fig. 18. The low ECC value attained by the antenna, and the steady omnidirectional radiating properties of each element, directly contribute to this ideal DG. This can decrease multipath fading and improve link reliability in a multipath vehicular environment.

6 $$DG=10\sqrt{1-{{\rho}_{e}}^{2}}$$

**Fig. 19:**
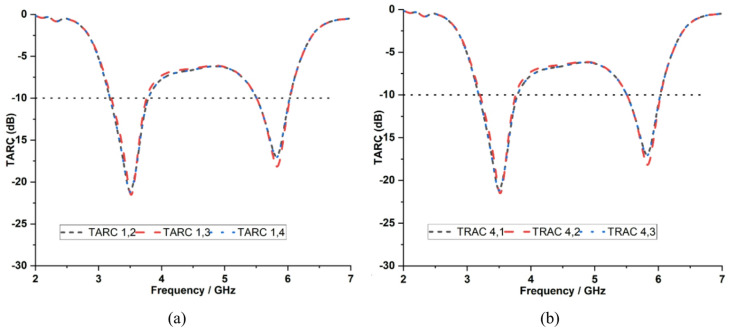
Measured TARC characteristics with respect to **a** Port 1, **b** Port 4.

TARC serves as an indicator of the diversity array efficiency and reflects the impact of mutual coupling between antenna elements. It is determined using Eq. (7). The measured TARC values for the proposed antenna remain below −10 dB at 3.5 GHz and 5.9 GHz ensuring stable operation and strong isolation among the antenna elements as shown in Fig. 19.

7 $$TARC=\sqrt{\frac{\left({\left|\right({S}_{11}+{S}_{12}{e}^{j\theta}\left)\right|}^{2}+{\left|\right({S}_{21}+{S}_{22}{e}^{j\theta}\left)\right|}^{2}\right)}{2}}$$

**Fig. 20:**
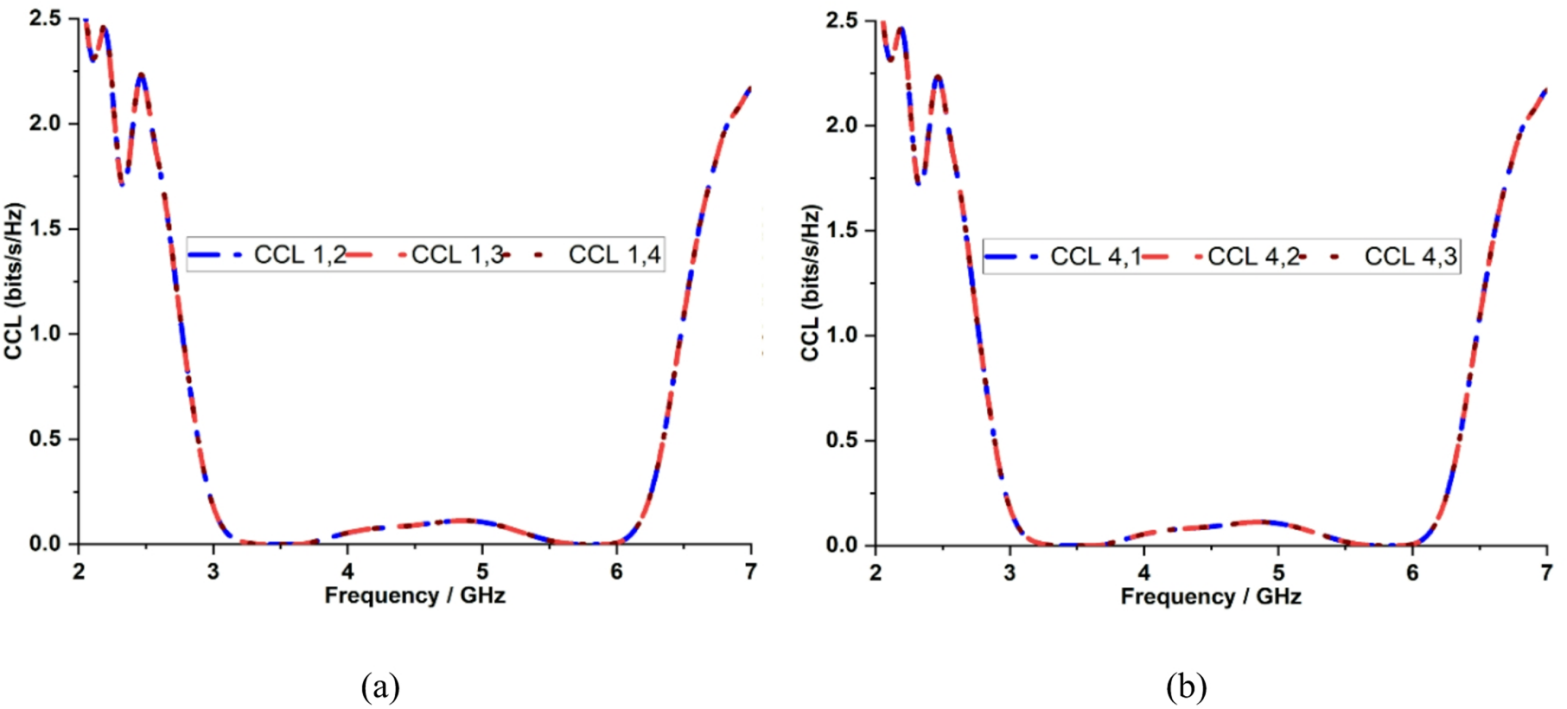
Measured CCL characteristics with respect to **a** Port 1, **b** Port 4.

To assess the impact of mutual coupling on data rates, Eqs. (8) and (9) are used to calculate the channel capacity loss (CCL) of the proposed quad-port MIMO antenna. The measured CCL is approximately 0.1 bits/s/Hz at both operating bands of 3.5 GHz and 5.9 GHz, as depicted in Fig. 20, which demonstrates an efficient data transmission capability with high isolation and low correlation without the need for extra decoupling structures, directly result in low CCL.

8 $$CCL=-log\left|{\psi}^{R}\right|$$

9 $$\left|{\psi}^{R}\right|=\left(\begin{array}{cc}{\rho}_{11}&{\rho}_{12}\\{\rho}_{21}&{\rho}_{22}\end{array}\right)$$

**Table 2:** Comparison of existing MIMO antennas with the proposed antenna.

Ref., Year	Antenna Size (λ_g_ × λ_g_)	Bands (GHz)	BW (MHz)	Iso. (dB)	Gain (dBi)	ECC	CCL (bps/Hz)	Profile/Volume	Fabrication Complexity	Vehicular/Housing Validation
^9^, 2024	1.1 × 1.1	3.5/5.9	500/1200	> 22	3.9/4.8	0.006	NA	Planar, single-layer (0.8 mm)	Moderate (strips + DGS)	No
^20^, 2018	2.14 × 1.16	3.5/5.9	450/1080	> 12	NA	< 0.15	NA	Large, high-profile array (0.8 mm)	High (8 × 8, parasitic, DGS)	No
^26^, 2023	1.22 × 1.22	3.5/5.9	870/1320	> 19	1.2/3.2	< 0.002	0.4	Planar (0.8 mm)	Moderate (geometry optimization)	No
^27^, 2023	0.68 × 0.68	3.5/5.9	910/2240	> 23	2.4/4.5	< 0.064	0.06	Planar (0.8 mm)	Moderate (stubs + DGS)	No
Prop.	0.95 × 0.95	3.5/5.9	680/670	> 20	3.6/4.7	< 0.03	0.1	Low-profile, single-layer (0.13 mm)	Low (no vias/SIW/CSRR/NL)	Yes (housing + vehicle rooftop CAD)

Table 2 summarizes recent advancements that highlight the comparative performance of the proposed quad-port dual-band antenna, demonstrating superior characteristics across various performance metrics.

- The proposed antenna structure maintains a compact geometry relative to the antenna designs presented in^9,20,26^.
- The proposed antenna has a compact low-profile single-layer design (0.13 mm thickness) in contrast to the high-profile designs described in^9,20,26^, and^27^.
- The proposed antenna structure effectively suppresses mutual coupling between ports, with isolation levels exceeding 20 dB without employing additional decoupling structures.
- The proposed antenna structure achieves higher gains of 3.6 dBi and 4.7 dBi at 3.5 GHz and 5.9 GHz compared to antennas reported in^9,20,26,27^.
- The proposed antenna structure improves ECC performance compared to designs in^20,27^.
- The proposed antenna maintains a low CCL of 0.1 bits/s/Hz compared to^26^, indicating minimal capacity degradation and effective utilization of spatial diversity.
- The proposed quad-port dual-band antenna at 3.5 GHz (680 MHz bandwidth) and 5.9 GHz (670 MHz bandwidth) meets the minimum bandwidth requirement of 5G NR78 band of 100 MHz^32^ and 75 MHz of DSRC in reference to V2X communication^33^. It has a compact structure and maximum gain compared to other works in^9,20,26,27^.
- Although the majority of the published antennas in^9,20,26^, and^27^ are assessed at the condition of free space only, the proposed structure is validated under realistic vehicular scenario by incorporating housing effect and full vehicle rooftop CAD analysis to account of vehicular body induced effects. Consequently, the antenna has stable impedance characteristics and identical radiation properties. This ascertains its strong applicability to real 5G-V2X uses.

In comparison to other existing designs based on SIW cavities, CSRRs, neutralization lines, parasitic elements or multilayers. The proposed design significantly reduces structural complexity, fabrication tolerance issues and overall antenna footprint compared with reported designs in^2,9,20,23,26^.

### Investigation of the proposed dual-band antenna on vehicle body

**Fig. 21:**
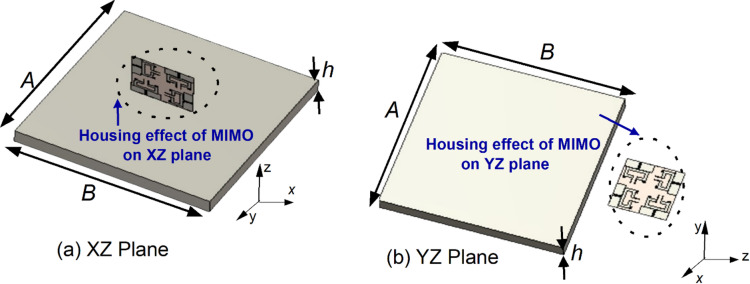
Proposed MIMO antenna: Housing effects.

In V2X application, antennas may be integrated on side mirror, roof top or embedded within the vehicular internal circuitry. Mounting the antenna on the vehicular body may change the characteristics of antenna performance. Several factors may cause changes in the antenna characteristics, such as impedance bandwidth and radiation patterns, due to the objects located nearby or large conducting parts of the vehicular body. The proposed antenna is mounted in two different locations on the vehicle to investigate the effect of antenna characteristics on the housing^34^. Figure 21 depicts the mounting of proposed dual-band MIMO antenna on the XZ and YZ planes of a large metal body, taking into account the roof of the vehicle. To facilitate vehicular communication, the roof top of the vehicle is chosen for various reasons, such as securely installing antennas without affecting the aerodynamics, high diversity gain, minimum multipath fading, antenna to have minimal obstruction to its impedance bandwidth and omnidirectional radiation for V2X application^4,16^.

The proposed quad-port dual-band MIMO antenna is simulated with various sizes of (*A* × *B* × *h*) metal plates. At first, the proposed MIMO antenna is fixed vertically (XZ plane) 10 mm above the conducting ground plane, as shown in Fig. 21(a). Second, the proposed MIMO antenna is fixed horizontally (YZ plane) 25 mm away from the same ground plane, as shown in Fig. 21(b). Placing the antenna directly on the large metal plate influences frequency rejection on the desired band^34^, therefore the antenna is located above the conducting plate at the height mentioned above on the XZ and YZ planes. To understand the housing effect on impedance bandwidth and radiation performance, the sizes of metal plates are changed from 200 mm × 200 mm × 50 mm to 800 mm × 800 mm × 50 mm on each plane.

**Fig. 22:**
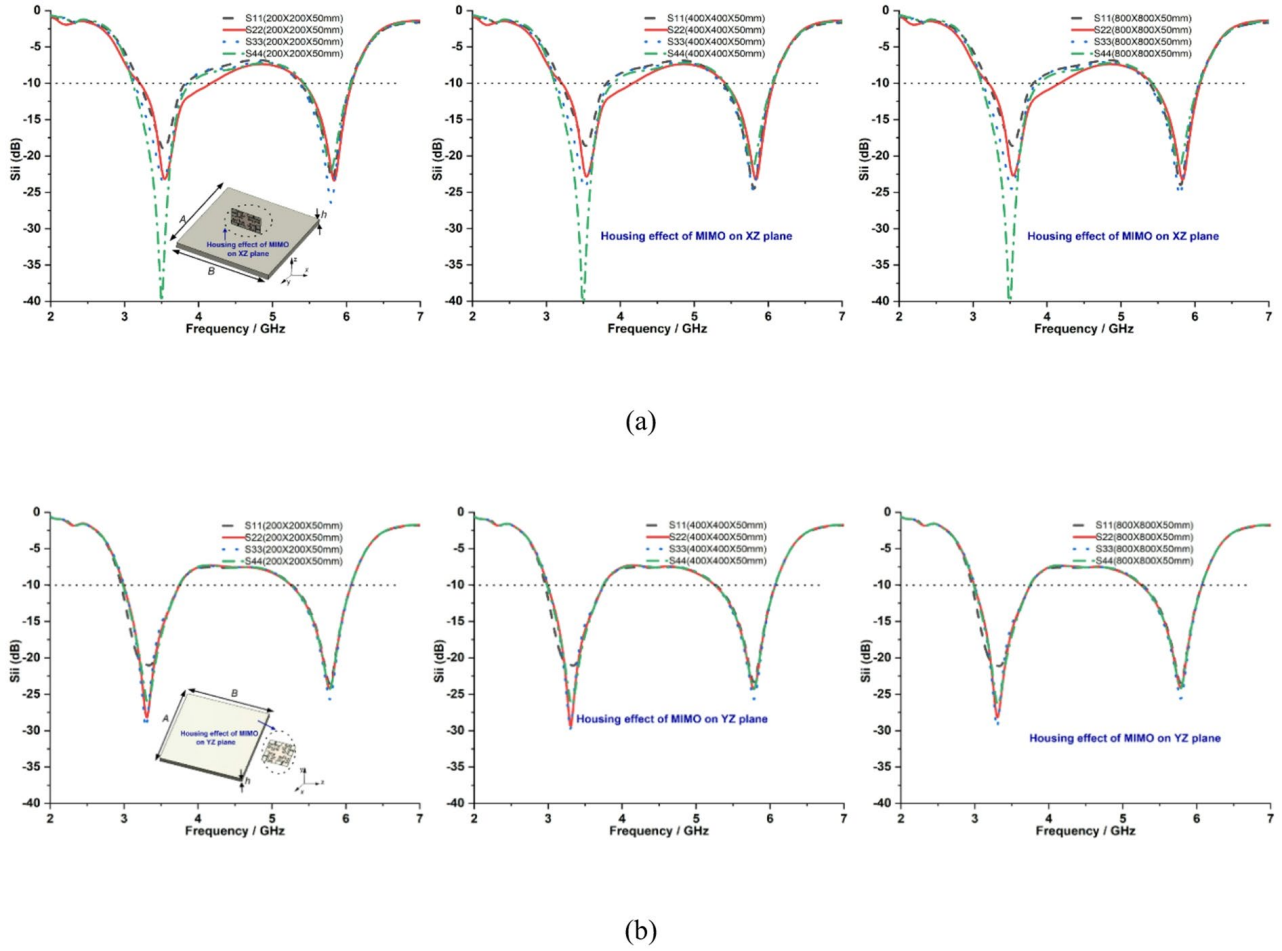
Reflection coefficients of proposed MIMO antenna on different size metal plates when the housing effect is considered: **a** XZ plane, **b** YZ plane.

**Fig. 23:**
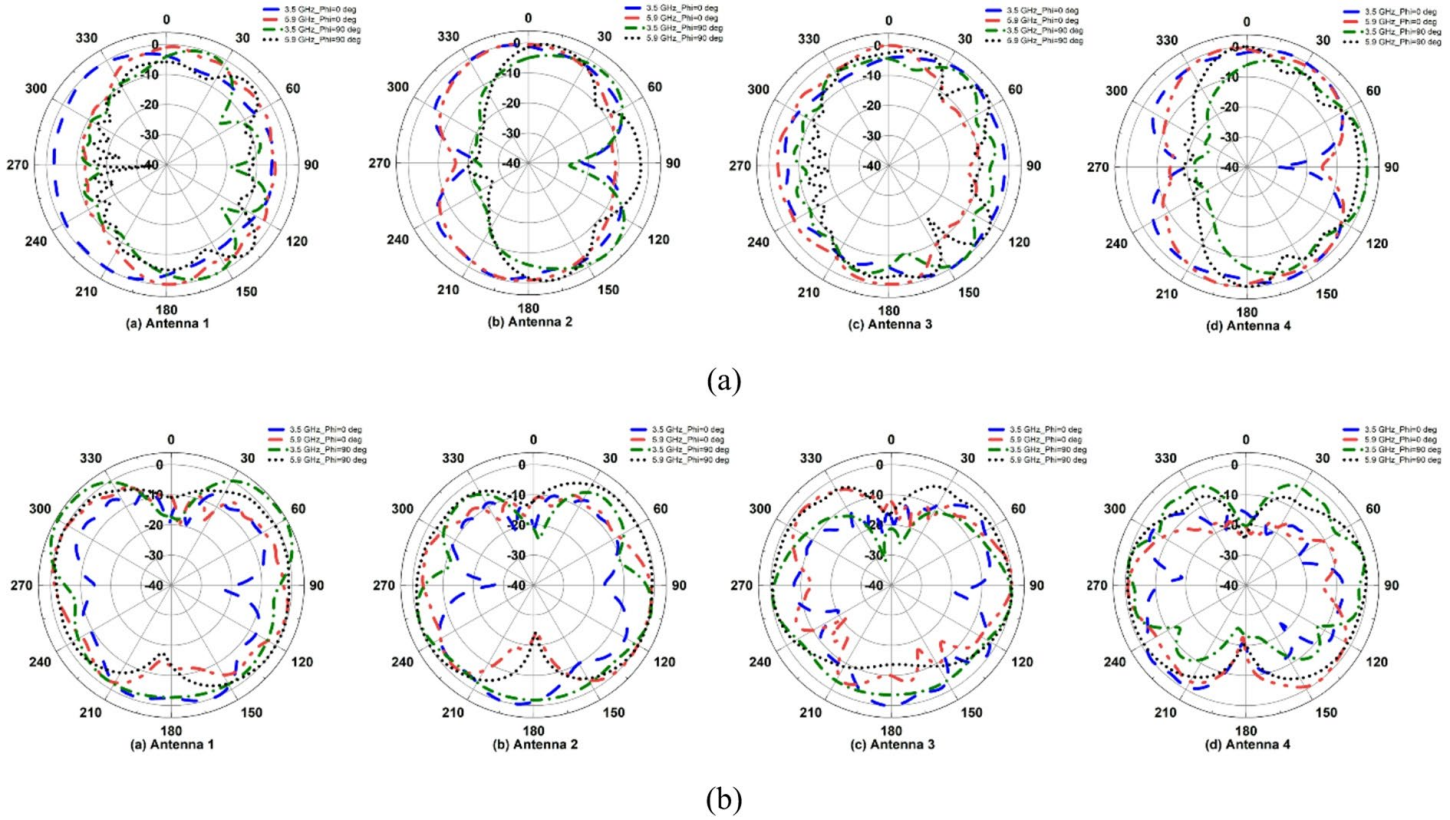
Far-field characteristics of proposed MIMO antenna when the housing effect is considered: **a** Antenna placed in the XZ plane (Size: 800 mm × 800 mm × 50 mm), **b** Antenna placed in the YZ plane (Size: 800 mm × 800 mm × 50 mm).

Figures 22 and 23 show the simulated reflection coefficients and far-field characteristics of the proposed MIMO antenna under the housing effect on the XZ plane and YZ plane, respectively. It is observed that the different size of conducting plate has less influence on the proposed antenna impedance bandwidth coverage on both scenarios, as shown in Fig. 22. The large conducting surface directs energy at right angles, resulting in no signals in the far-field patterns of the proposed MIMO antenna due to diffraction. This increases directivity by ∼3 dB, as shown in Fig. 23. As a result, the ability of the antenna to direct energy toward the surrounding environment is not affected by its height above the conducting plate.

**Fig. 24:**
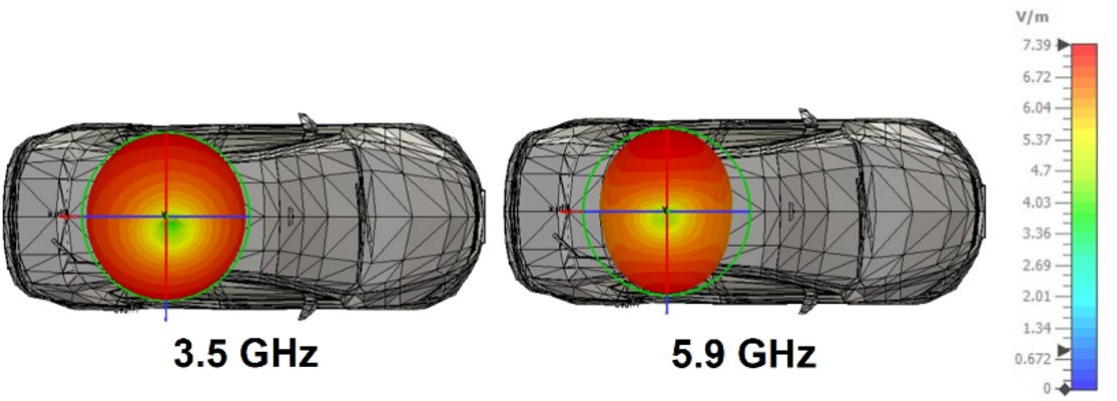
Investigation of proposed antenna on car body (3D model).

Figure 24 presents the analysis of the far-field radiation pattern for the designed dual-band antenna installed on a vehicle. The vehicle roof top is selected as the antenna mounting location to investigate the impact of the vehicular structure on its far-field radiation performance. The antenna demonstrates an almost omnidirectional radiation behaviour at 3.5 GHz and 5.9 GHz. The broad metallic surface on the vehicle roof contributes to improved antenna directivity, with roof-induced signal reflections functioning as an additional ground plane. When the antenna is positioned on the conductive surface, electromagnetic waves reflected off the roof appear to be coming from an extended ground plane. This expanded ground surface reduces unwanted back radiation, boosts forward directed radiation and increases the overall directivity of the antenna. In addition, the metallic roof stabilizes the radiation pattern and eliminates pattern distortion, which is common when antennas interact with complicated vehicle features.

## Conclusion

A compact quad-port dual-band MIMO antenna has been designed, fabricated and validated for 5G (n78) and V2X (DSRC) applications. The proposed configuration with multi-stub radiating structure and a defected ground, enhanced the impedance matching and bandwidth. Measured results confirm stable operation at 3.5 GHz and 5.9 GHz with impedance bandwidth of 680 MHz and 670 MHz by keeping mutual coupling below −20 dB. The antenna exhibits a peak gain of 3.6 dBi and 4.7 dBi at the respective bands, maintaining an efficiency above 83%. Diversity analysis yields ECC < 0.03, DG ≈ 9.99 dB, TARC < −10 dB and CCL ≈ 0.1 bits/s/Hz, indicating maximum isolation and diversity performance without employing any additional decoupling structures such as SIW or CSRR. When mounted on a vehicle rooftop, the antenna maintains an omnidirectional radiation pattern with improved directivity due to the metallic surface effect. Compared with reported designs, the proposed antenna offers a favourable trade-off between compactness, gain and diversity metrics, making it a strong candidate for integration in next-generation 5G-enabled vehicular communication system.

## Data Availability

The datasets used and/or analyzed during the current study available from the corresponding author on reasonable request.
